# Incidence of anemia and red blood cell (RBC) transfusion requirement in breast cancer

**DOI:** 10.22088/cjim.14.2.237

**Published:** 2023

**Authors:** Nur Aklina Ramli, Salfarina Iberahim, Ahmad Arif Che Ismail, Hasmah Hussin

**Affiliations:** 1 Department of Clinical Medicine, Advanced Medical and Dental Institute, Universiti Sains Malaysia, 13200 Kepala Batas, Penang, Malaysia; 2 Department of Haematology and Transfusion Medicine Unit, School of Medical Sciences, Universiti Sains Malaysia Health Campus, 15200 Kubang Kerian, Kelantan, Malaysia; 3 Blood Bank Unit, Hospital Raja Perempuan Zainab (II), 15586 Kota Bharu, Kelantan, Malaysia

**Keywords:** Breast cancer, Anaemia, Red cell transfusion, Chemotherapy

## Abstract

**Background:**

Cancer-related anaemia is one of the main burdens in oncology, although the available data on its prevalence and treatment options such as blood transfusion are often contradictory. This study aimed to evaluate the prevalence of anaemia and the requirement for packed red blood cell (PRBC) transfusion among women with breast cancer (BC) and to determine the associated factors for chemotherapy-induced anaemia (CIA).

**Methods:**

This cross-sectional retrospective study conducted in Kelantan involved 104 newly diagnosed female BC patients from 2015 to 2016 who underwent chemotherapy. For statistical analysis, chi-square was used to compare between CIA and non-CIA groups. In addition, simple and multiple logistic regression were used to determine the association of the CIA.

**Results:**

Our study revealed that 34.6% (n=36) of patients had mild anaemia, and 59.6% (n=62) had normal haemoglobin at pre-chemotherapy. The prevalence of anaemia increased from 40.4% to 77% at the end of our study. About 30.8% of patients received PRBC transfusion during chemotherapy with mean haemoglobin before the first transfusion of 7.9 g/dl. CIA was observed in 54.8% of cases. There was no significant association between CIA concerning the patient characteristic, cancer characteristic, or cancer treatment.

**Conclusion:**

We concluded that a significant proportion (40.4%) of BC patients was anaemic even before chemotherapy, with the red blood cell requirements up to 30.8% throughout chemotherapy. A larger prospective study is needed to determine the predictors for the CIA and subsequently improve patient management.

Although Anaemia and associated symptoms are significant health problems among oncology patients worldwide. Anaemia is usually discovered among breast cancer (BC) patients at initial diagnosis or following chemotherapy, which directly influences disease progression. Anaemia can result from multiple causes either related to the direct tumour invasion, its treatments (radiation) or chemotherapy-induced anaemia (CIA). The main mechanism is reducing erythropoietin (EPO) level secondary to nutritional deficiencies or bone marrow injury due to metastases or myelosuppressive chemotherapy (1). Although the management of anaemia during chemotherapy remains challenging, analysing the prevalence of anaemia and its contributing factors are crucial. Different approaches for anaemia were discussed include the use of erythropoiesis-stimulating agents (ESAs), iron therapy or blood transfusion (2). 

Additionally, transfusion of RBC is considered as a rapid correction of the haemoglobin level in patients with symptomatic anaemia. Despite the presence of clinical transfusion guidelines for the treatment of anaemia among cancer patients, a variation of PRBC transfusion practice still exists among clinicians. Hence, local data are required to further improve blood transfusion safety. The study aimed to determine the prevalence of anaemia, the requirement for red blood cell transfusion, and the associated factors for anaemia among BC patients who underwent chemotherapy in the two tertiary centres. These findings can help us plan for future interventions in managing anaemia, promoting appropriate and safe transfusion practices. Optimising the care is essential to minimise the adverse consequences and subsequently improve patient outcomes.

## Methods

**Study population and methods: **This cross-sectional study involved 104 female BC patients from Hospital Universiti Sains Malaysia (HUSM) Kubang Kerian and Hospital Raja Perempuan Zainab HRPZ (II), Kota Bharu, Kelantan from January 2015 to December 2016. Patients with benign breast disease, underlying end-stage renal failure on regular haemodialysis, history of inherited bleeding disorders or haemoglobinopathy, pregnancy at the time of the diagnosis, requiring anticoagulant or antiplatelet, or patients below 18 years were excluded. A subject who met the criteria of inclusion was chosen randomly until the required sample size was achieved.

The demographic features, cancer characteristics, cancer treatment, laboratory investigations and PRBC transfusion were collected retrospectively. The pre-chemotherapy variables were extracted from the patient records such as age, race, underlying co-morbidities, stage of cancer, histological grade, histopathological features, oestrogen receptor (ER), progesterone receptor (PR), and human epidermal growth factor receptor 2 (HER2) status. The regimens of chemotherapy and surgical intervention or radiotherapy and hormonal treatment received were also recorded. The haemoglobin (Hb) level was determined before and after chemotherapy. Anaemia is defined as a Hb level less than 12 g/dl in non-pregnant women by World Health Organization (WHO). According to the common toxicity criteria of the National Cancer Institute (NCI) and the European Organization for Research and Treatment of Cancer (EORTC), the severity of anaemia is further categorised as mild anaemia: Hb = 11.9 g/dl - 10 g/dl, moderate anaemia: Hb = 9.9 g/dl - 8 g/dl, and severe anaemia: Hb < 8.0 g/dl. Chemotherapy was given to all patients with the following regimens: FEC (Fluorouracil, Epirubicin, Cyclophosphamide) or TAC (Docetaxel, Adriamycin, Cyclophosphamide) or AC (Adriamycin, Cyclophosphamide). The subjects were followed up until the completion of the sixth cycle of chemotherapy. Patients with normal haemoglobin before chemotherapy and developed anaemia after chemotherapy or patients who had worsening severity of anaemia post-chemotherapy were defined as chemotherapy-induced anaemia.

**Ethical consideration: **We obtained ethical approval from Human Ethics Committee at the Hospital Universiti Sains Malaysia with the code of ethics of USM/JEPeM/19120949 and the Medical Research and Ethics Committee (MREC) Ministry of Health Malaysia with the code of ethics of NMRR-19-3471-51818. The confidentiality of the subjects was strictly protected.

**Statistical analysis: **Data were analysed with Statistical Package for Social Sciences (SPSS) software version 26.0 for window-software (SPSS, Chicago, Illinois, USA). A Chi-Square test was performed to compare the differences between CIA groups and without CIA groups for each discrete variable. Factors associated with the prevalence of CIA, such as patients’ characteristics, cancer characteristics or cancer treatment, were analysed using univariable linear regression. The association of CIA was then adjusted for other variables using multiple linear regression (MLR) to identify any statistically significant risk factors between CIA and potential predictive variables. P values below 0.05 were considered statistically significant. 

## Results

The patients’ and cancer characteristics were summarised in Table 1. More than half of the studied patients were aged 50 years old and above (n=57, 54.8%), while 45.2% (n=47) were aged below 50 years old. The minimum age of the patient was 25 years old, and the maximum age was 84 years old. Most patients had no underlying medical illness (n=63, 60.6%). Meanwhile, 24 (23.1%), 4 (3.8%) and 10 (9.6%) of them had hypertension, diabetes mellitus or both, respectively. Stage IV BC was the most predominant group (n=38, 36.5%), followed by stage III (n=37, 35.6%), stage II 

 (n=25,24%) and stage I (n=4,3.9%). Out of 103 patients who underwent surgery, 71.1% (n=74) was performed before chemotherapy, whereas 26% (n=27) of patients had an operation after chemotherapy. The majority of patients received radiotherapy after completing the chemotherapy (n= 74, 71.2%), whereas 24% (n=25) of them never received any radiotherapy.

 Meanwhile, 79.8% (n=83) of patients received hormonal therapy throughout cancer treatment. Overall, 51.9% (n=54) of patients received FEC (Fluorouracil, Epirubicin, Cyclophosphamide), 42.3% (n=44) received TAC (Docetaxel, Adriamycin, Cyclophosphamide) and 5.8% (n=6) received AC (Adriamycin, Cyclophosphamide) as the chemotherapy regimen. About 62 patients (59.6%) had baseline Hb within the normal range before the initiation of the chemotherapy. Mild anaemia was observed in 34.6% of patients (n=36), and 4.8% (n=5) had moderate anaemia. Only one patient was documented with severe anaemia (1%) (Table 2). The baseline Hb level ranged from 7.9 g/dl to 14.8 g/dl with mean Hb of 12.1 g/dl. Majority of them had normocytic normochromic red cells (83.7%, n=87), whereas microcytic hypochromic features were demonstrated in 17 patients (16.3%) (Table 3). Out of the total of 104 subjects, non- transfused patients were 69.2% (n=72) compared to the transfused group of 30.8% (n= 32). From the transfused group of 32 patients, 53.1% (n=17) received one unit of RBC transfusion and 40.6% (n=13) were transfused with two units of RBC (Table IV). No incidence of acute adverse transfusion reaction was experienced during or post-transfusion. CIA was reported in 57 patients (54.8%), whereas 45.2% (47 patients) had Hb levels within normal range or not worsening anaemia post-chemotherapy. There was no significant difference observed in the distribution of patients’ characteristics, cancer characteristics, or cancer treatment between chemotherapy and non- CIA groups (table 5). Factors associated with the prevalence of CIA such as the patient's age, race, underlying co-morbidities, baseline Hb, and RBC indices (MCV, MCH) were analysed. The analysis concluded that all these variables were not clinically significant for the patients’ characteristic association with anaemia status (table 4). The group of patients who developed CIA was found to be insignificant with the cancer stage (p=0.847), histopathological features (p=0.748), or tumour grade (p=0.065). The receptor status of the patients, such as ER, PR and HER2 in general, did not show any significance towards the CIA. There was no significance between the CIA in terms of initial treatment with radiotherapy (p=0.414), surgical intervention (p=0.538) or hormonal treatment (p=0.222). No significant difference between types of chemotherapy regimen with CIA was observed (p=0.926) (table 6) in SLR. 

A p-value less than 0.25 is supported by the literature to be included in multivariable analysis (3). After adjusting each confounding factor with race, baseline haemoglobin, histological tumour grade, HER2 receptor status, and hormonal treatment, CIA remained insignificant in the MLR analysis (table 7). 

**Table 1:** Patient’s characteristics among breast cancer patients (n=104)

**Variables**	**n (%)**
**Patient characteristics** ***Age, years***	
**< 50 years**	47 (45.2)
**≥ 50 years**	57 (54.8)
***Race***	
**Malay**	93 (89.4)
**Chinese**	9 (8.7)
**Others**	2 (1.9)
***Comorbid***	
**None**	63 (60.6)
**Hypertension or Diabetes mellitus or Asthma**	31 (29.8)
**Hypertension and Diabetes mellitus**	10 (9.6)
**Stage of tumour**	
** Stage I**	4 (3.9)
** Stage II**	25 (24.0)
** Stage III**	37 (35.6)
** Stage IV**	38 (36.5)
***Histological tumour grade***	
** Grade I**	23 (22.1)
** Grade II**	56 (53.9)
** Grade III**	25 (24.0)
***Histopathological features***	
** Invasive ductal carcinoma**	94 (90.4)
** Invasive lobular Carcinoma**	10 (9.6)
***Estrogen Receptor (ER) status***	
** Negative** ** Positive**	34 (32.7) 70 (67.3)
***Progesterone Receptor (ER) status***	
** Negative** ** Positive**	49 (47.1) 55 (52.9)
*HER2 receptor	
**Negative** **Positive**	48 (46.2) 56 (53.8)
***Surgery***	
** Yes**	
** Before chemotherapy**	74 (71.1)
** After chemotherapy**	27 (26.0)
** No**	3 (2.9)
***Radiotherapy***	
** None**	25 (24.0)
** Before chemotherapy**	5 (4.8)
** After chemotherapy**	74 (71.2)
***Hormonal therapy***	
** No** ** Yes**	21 (20.2) 83 (79.8)
***Types of chemotherapy***	
**FEC (Fluorouracil, Epirubicin, Cyclophosphamide)**	54 (51.9)
**TAC (Docetaxel, Adriamycin, Cyclophosphamide)**	44 (42.3)
**AC (Adriamycin, Cyclophosphamide)**	6 (5.8)

*HER2 receptor – human epidermal growth factor receptor 2

**Table 2:** Pre-chemotherapy haemoglobin level of breast cancer patients

**Haemoglobin level**	**n (%)**
**Normal haemoglobin (Hb 12 -16 g/dl)**	62 (59.6)
**Mild anaemia (Hb 10-11.9 g/dl)**	36 (34.6)
**Moderate anaemia (Hb 8-9.9 g/dl)**	5 (4.8)
**Severe anaemia (Hb < 8 g/dl)**	1 (1.0)

**Table 3:** Pre-chemotherapy red blood cell indices of breast cancer patients

**Range of MCV and MCH**	**n (%)**
**Normocytic normochromic** **MCV: ** **80-100 fL** **MCH: 27-32 pg**	87 (83.7)
**Microcytic hypochromic** **MCV < 80 fL** **MCH < 27 pg**	17 (16.3)

**Table 4:** Transfusion of red blood cells among breast cancer patients during chemotherapy (n=104)

**Variables**	**n (%)**
***RBC transfusion***	
Yes	32 (30.8)
No	72 (69.2)
***Total number of RBC transfusion***	
1	17 (53.1)
2	13 (40.6)
>2	2 (6.3)

**Table 5:** Patient characteristics, cancer characteristics and cancer treatment between chemotherapy-induced anaemia and non-chemotherapy-induced anaemia groups (n=104)

	**Chemotherapy-induced anaemia (CIA)**				**Chi-square**	***p*** **-value**
	**Yes ** **n (%)**		**No** **n (%)**			
**Age**					2.81	0.093
**<50 years**	30 (52.6)		17 (36.2)			
**≥ 50 years**	27 (47.4)		30 (63.8)			
**Race**					4.49	0.105
**Malay**	54 (94.7)		39 (83.0)			
**Chinese**	3 (5.3)		6 (12.8)			
**Others**	0 (0.0)		2 (4.2)			
**Comorbid**					1.03	0.597
**None**	35 (61.4)		28 (59.5)			
**One**	18 (31.6)		13 (27.7)			
**Two**	4 (7.0)		6 (12.8)			
**Stage of tumour**					0.854	0.836
**I**	3 (5.3)		1 (2.1)			
**II**	14 (24.6)		11 (23.4)			
**III**	19 (33.3)		18 (38.3)			
**IV**	21 (36.8)		17 (36.2)			
**Histological tumour grade**					5.66	0.059
**I**	12 (21.0)		11 (23.4)			
**II**	36 (63.2)		20 (42.6)			
**III**	9 (15.8)		16 (34.0)			
**Histopathological features**					0.10	0.752
**Invasive ductal carcinoma**	52 (91.2)		42 (89.4)			
**Invasive lobular carcinoma**	5 (8.8)		5 (10.6)			
**Estrogen Receptor (ER) status**					1.22	0.299
**Negative** **Positive**	16 (28.1) 41 (71.9)		18 (38.3) 29 (61.7)			
**Progesterone Receptor (PR)status**					1.27	0.260
**Negative** **Positive**	24(42.1) 33(57.9)		25(53.2) 22(46.8)			
*HER2 receptor					1.70	0.191
**Negative** **Positive**	23(40.1) 34(59.6)		25(53.2) 22(46.8)			
**Variables**	β		95 % CI			
**Estrogen Receptor (ER) status ** **Negative** **Positive**	1.591		0.69- 3.62			
**Progesterone Receptor (PR) status** **Negative** **Positive**	1.562		0.71-3.40			
*HER2 status **Negative** **Positive**	1.680		0.77- 3.66			
**Prior surgery** **No** **Yes**	0.850		0.68-10.61			
**Prior radiotherapy** **No** **Yes**	0.704		0.28- 1.76			
**Hormonal treatment** **No** **Yes**	1.829		0.69- 4.81			
**Types of chemotherapy** **FEC** **TAC** **AC**	1.134 0.862		0.50- 2.52 0.15- 4.66			

*HER2 receptor – human epidermal growth factor receptor 2

**Table 6:** Associated factors for chemotherapy-induced anaemia by multiple logistic regression (n=57)

	**Chemotherapy-induced anaemia (CIA) **		**Chi-square**	***p*** **-value**
	**Yes** **n (%)**	**No** **n (%)**		
**Age**			2.81	0.093
<50 years	30 (52.6)	17 (36.2)		
≥ 50 years	27 (47.4)	30 (63.8)		
**Race**			4.49	0.105
Malay	54 (94.7)	39 (83.0)		
Chinese	3 (5.3)	6 (12.8)		
Others	0 (0.0)	2 (4.2)		
**Comorbid**			1.03	0.597
None	35 (61.4)	28 (59.5)		
One	18 (31.6)	13 (27.7)		
Two	4 (7.0)	6 (12.8)		
**Stage of tumour**			0.854	0.836
I	3 (5.3)	1 (2.1)		
II	14 (24.6)	11 (23.4)		
III	19 (33.3)	18 (38.3)		
IV	21 (36.8)	17 (36.2)		
**Histological tumour grade**			5.66	0.059
I	12 (21.0)	11 (23.4)		
II	36 (63.2)	20 (42.6)		
III	9 (15.8)	16 (34.0)		
**Histopathological features**			0.10	0.752
Invasive ductal carcinoma	52 (91.2)	42 (89.4)		
Invasive lobular carcinoma	5 (8.8)	5 (10.6)		
**Estrogen Receptor (ER) status**			1.22	0.299
Negative Positive	16 (28.1) 41 (71.9)	18 (38.3) 29 (61.7)		
**Progesterone Receptor (PR)status**			1.27	0.260
Negative Positive	24(42.1) 33(57.9)	25(53.2) 22(46.8)		
*HER2 receptor			1.70	0.191
Negative Positive	23(40.1) 34(59.6)	25(53.2) 22(46.8)		
**Variables**	**β**	**95 % CI**	**p-value**	
**Estrogen Receptor (ER) status** Negative Positive	1.591	0.69- 3.62	0.270	
**Progesterone Receptor (PR)status** Negative Positive	1.562	0.71-3.40	0.261	
*HER2 status Negative Positive	1.680	0.77- 3.66	0.192*	

*Her2 receptor – human epidermal growth factor receptor 2

**Table 7:** Associated factors for chemotherapy-induced anaemia by multiple logistic regression (n=57)

**Variables**	**β**	**95 % CI**	***p*** **-value**
**Race** Chinese, Others Malay	0.331	0.06- 1.65	0.405
**Baseline haemoglobin** Normal Mild anaemia Moderate anaemia Severe anaemia	0.439 0.148	0.17- 1.09 0.01- 1.62	0.137
**Histological tumour grade** I II III	1.660 0.487	0.56 -4.84 0.12 -1.86	0.990
*HER2 Negative Positive	1.025	0.34- 3.07	0.964
**Hormonal treatment** No Yes	1.039	0.25- 4.32	0.958

β: regression coefficient. Forward and backward (likelihood ratio) methods were

applied. p < 0.05 taken as significant value at 95% confidence interval (CI).

Hosmer–Lemeshow test, p-value = 0.351.Classification table overall percentage

correct = 69.2%.

*HER2 receptor – human epidermal growth factor receptor 2

## Discussion

**Prevalence of anaemia before the chemotherapy:** Although anaemia is not a commonly encountered symptom among BC, a group of patients can be anaemic at the time of diagnosis. It could be discovered incidentally during blood investigations before any surgical intervention or chemotherapy. The prevalence of pre-chemotherapy anaemia varied significantly between many studies, attributed to the variation in the patients’ characteristics, the clinical spectrum of disease, or treatment modalities. We also need to consider that patients may be presented late for treatment. As a result, anaemia can worsen at the time of initial diagnosis. Thus, increasing awareness with early screening and detection of BC is essential. 

Overall, there is a significant proportion, 40.4% of BC patients were anaemic when the initiation of chemotherapy. Our prevalence of anaemia was almost equivalent to the findings by Pourali *et al.,* who reported the rate of prechemotherapy anaemia was 41%. However, the result may vary depending on the studied population. Although most of our patients had no underlying co-morbidities, most of them presented with the advanced stage of cancer (4). On the contrary, a study by Nair *et al. *demonstrated that pre-chemotherapy anaemia was as high as 60% among BC patients with 41.6% having mild anaemia (5). The different prevalence of anaemia compared to this study was possibly contributed by a variation of anaemia among the female population, which differed between the countries. A cross-sectional study of solid tumours mainly involved BC patients reported mild anaemia in 59.1% of their patients before chemotherapy, and their patients were further classified into anaemia of chronic disease or iron deficiency anaemia based on their laboratory parameters such as serum iron, serum ferritin and C - reactive protein (CRP) (6). Surprisingly, only 7.1% (3/42) of anaemic patients in our study had iron studies recorded. Therefore, we did not further stratify patients because these investigations were not routinely performed. Nevertheless, the clinicians need to identify this treatable cause of anaemia before chemotherapy and commencing the iron supplementation even before patients are truly deficient. Hence, routine iron studies are suggested to be performed before the first cycle of chemotherapy. 

Another study by Zhu and colleagues concluded that 25.3% were anaemic before the chemotherapy, which was lower than this study. The disparity may be related to the fact that we had more patients aged 50 years old and above (54.8% versus 39.4%), and their study involved locally advanced BC (7). A study by Jeffery *et al.* concluded 31.3% were anaemic pre-chemotherapy in stage II and III BC patients treated with adjuvant chemotherapy (8). A European Cancer Anaemia Survey (ECAS) also demonstrated a lower prevalence of anaemia at the time of enrolment (9). All of these studies had a larger sample size compared to us.

**Requirement for RBC transfusion** **:** Patients with cancer usually have transitory bone marrow failure that necessitates transfusion support. Generally, from our study, most patients required two or fewer PRBC transfusions throughout their chemotherapy except for two cases. Both patients were in stage IV BC and developed severe anaemia following the first cycle of TAC. All moderate and severe anaemia patients at the initial diagnosis were transfused following the first cycle of chemotherapy. In comparison, 47.2% (17/36) patients with mild anaemia pre-chemotherapy received PRBC throughout different chemotherapy periods. Patients with positive ER, PR status or treated with FEC were more likely to receive the blood transfusion. In terms of transfusion requirements, our data recorded 30.8% of patients were transfused during chemotherapy, which was slightly higher than other studies (5,8). As most of these patients were mildly anaemic throughout chemotherapy, PRBC transfusion was infrequently required. The lowest pretransfusion Hb level was 4.9 g/dl, whereas the highest was 10.7 g/dl. A wide gap in the Hb can be explained by the difference in the local transfusion policy and clinician preferences. Therefore, individual factors and anaemic symptoms should be considered even though Hb level can influence transfusion decisions. As reported by Granfortuna *et al.*, fatigue is the main indication for PRBC transfusion in non-myeloid cancer patients, followed by dyspnoea on exertion and pallor (10). 

Another study also supported a statistically significant improvement in fatigue and breathlessness among palliative BC patients after blood transfusion (11). Therefore, PRBC transfusion can provide a rapid response to anaemia related symptoms, improving patients’ overall performance and quality of life. Out of 32 patients with transfusion history during chemotherapy, 7 (21.8%) patients were transfused at the Hb less than 7 g/dl, while 25 (78.1%) patients had a transfusion at the Hb of 7 g/dl or above. The mean Hb concentration was 7.9 g/dl before the first PRBC transfusion. This pattern was slightly inconsistent with the current guidelines for clinical use of blood by the National Blood Centre Ministry of Health Malaysia, as the Hb level around 7- 8 g/dl is appropriate to control symptomatic anaemia during the marrow-suppressive treatment of cancer (12). The National Comprehensive Cancer Network (NCCN) 2018 also recommended that PRBC transfusion in CIA, which Hb should be maintained more than 7 g/dl in asymptomatic stable patients or as needed for recovery of symptoms in patients with symptomatic anaemia (13).

Additionally, the one unit transfusion policy for the non-bleeding oncology patients was justifiable as 53.1% of our patients had a transfusion history with one pint of blood. This practice is in line with the International Society of Blood Transfusion (ISBT) guidelines that recommend a single unit transfusion followed by a clinical and laboratory evaluation to determine whether current transfusion therapy is appropriate or more units are required (14). Continuing education on single unit policy and regular audit with extensive feedback should be emphasised to the clinicians. For example, a choosing wisely initiative helps in increasing the proportion of one unit RBC transfusion through its implementation of computerised provider order entry (15). Furthermore, the impact on the clinical outcome between the liberal and restrictive groups among cancer patients was highlighted in few studies. A restrictive transfusion strategy was associated with reducing blood utilization without increasing perioperative morbidity and cancer-related mortality (16).

**Association between chemotherapy-induced anaemia and patient characteristics, cancer characteristics, and cancer treatment** **: **Our findings revealed CIA in 54.8% (57/104) of BC patients after completion of chemotherapy. However, the chi-square and logistic regression analysis findings showed an insignificant statistical association between the CIA with any variables. This finding contradicted the results of several studies (17, 18). The difference is reflected mainly by almost equal distribution between groups who developed CIA or not in this study. The variables also can be confounded by each factor in the multivariable analysis. Another possible explanation is the different definitions of CIA used in several studies. Jeffery and his colleagues defined an anaemic event as the drop of Hb to less than 10 g/dl, the use of blood transfusion or EPO therapy, whereas CIA in our study was defined as patients who develop anaemia from normal Hb or had worsening severity of anaemia post-chemotherapy (8).

The clinical variables of patients’ age, co-morbidities of the patients, cancer stages, and lower pre-chemotherapy haemoglobin are expected to be the substantial factors for CIA. Yet, we failed to observe any statistical significance between these variables with anaemia. Although our study included a large percentage of subjects with stage IV (36.5%) compared to a few patients in stage I (3.9%), higher cancer stages did not associate with risk for CIA. Furthermore, the results were evenly distributed between both groups at a similar stage VI (36.8% versus 36.2%, p = 0.854). Contrary to a multi-centre prospective study, their study indicated that advanced age and cancer stages were associated significantly with the severity of anaemia during chemotherapy (18). 

This study focused on participants with pre-chemotherapy Hb less than 12 g/dl and only a minority of them at stage VI. A validated prediction model for anaemia also demonstrated that low Hb concentration before a chemotherapy cycle and age 65 years or older were predictors of anaemia (19). As reported by Adeel and colleagues, there was a significant difference in Hb level between patients treated with docetaxel and cyclophosphamide (TC) compared to AC (doxorubicin and cyclophosphamide). Whereas, in this study, the different chemotherapy regimens did not result in significant anaemia (20). We observed that patients were equally treated between FEC or TAC and a minor percentage with AC, thus resulting in the myelosuppressive effect that was nearly equivalent. Hence, these findings reflected that a decline in Hb was expected among BC patients undergoing chemotherapy irrespective of their stages of cancer, age at diagnosis, pre-treatment Hb or types of chemotherapy or concomitant radiotherapy and hormonal treatment. As cancer patients are at risk of developing CIA, identification of mild anaemia is important to initiate oral iron supplement therapy to replenish the iron stores. A local study conducted by Bassam *et al.* regarding treatment patterns in solid cancer patients suggested starting iron supplements before anaemia develops and emphasizing the use of erythropoiesis-stimulating agents (ESAs) when Hb drops below 10 g/dl (21). In a study among oncology patients with moderate to severe iron deficiency anaemia, ferric carboxymaltose (FCM) administration resulted in a significant reduction in the transfusion requirements with a sustainable increase of Hb level (22). These transfusion alternatives can improve the haematopoietic response during chemotherapy while minimising the risk of allogeneic blood transfusion. Parental iron in combination with ESAs compared to ESAs alone for treatment of CIA have demonstrated a significant haematological response (23). Nevertheless, concerning the risk of thromboembolism and tumour progression, ESAs were only recommended for palliative patients with Hb less than 10 g/dl (24). Our study had several limitations. First, we were unable to investigate further other causes of anaemia such as nutritional deficiencies (iron, folic acid, and vitamin 12 deficiency) or acute blood loss, thus some anaemias can be undiagnosed. A comprehensive evaluation of anaemia including nutritional status, may help to guide anaemia treatment. Hence, we suggested iron studies as a standard pre-chemotherapy workup to identify patients with iron deficiency who can benefit from earlier administration of iron supplements. Our study also did not review different treatment patterns for anaemia and the long-term impact of anaemia on the quality of life and overall survival. Another limitation was the small sample size in this study. Selection bias may be present due to the retrospective nature of this study. The prevalence of anaemia in our study was relatively higher even before chemotherapy. However, our study found no remarkable association between patients’ characteristics, cancer characteristics or cancer treatment with the CIA. A large prospective study is warranted to evaluate patients who are most likely to develop anaemia, thereby allowing an earlier intervention to manage patients effectively. Additionally, our analysis demonstrated a significant group of patients were transfused throughout chemotherapy. Efforts can be made to enhance patient blood management (PBM), incorporate the use of transfusion alternatives and appropriate transfusion practices among clinicians. In addition, interventions such as iron therapy to optimize the haemoglobin level during chemotherapy should be considered.
